# Video-assisted thoracoscopic layered insertion of fibrin glue and polyglycolic acid sheet directly into ruptured bulla associated refractory secondary pneumothorax

**DOI:** 10.1186/1749-8090-8-S1-O300

**Published:** 2013-09-11

**Authors:** K Okabayashi, A Nagata, T Higuchi, T Hamada

**Affiliations:** 1 Department of General thoracic surgery, National Fukuoka-Higashi Medical Center, Fukuoka, Japan

## Background

Patients with secondary pneumothoraces generally have serious comorbid disease and poor pulmonary function and require that treatment be individualized. Though interventional treatments such as tube thoracostomy and pleurodesis are to be selected first, some cases become obstinate. Even though VATS treatment is attempted ultimately, sometimes it turned out to be inadequate for stapling or simple suturing. We present here our new VATS technique to control refractory air leak by direct infusion of fibrin glue (FG) via double lumen catheter and additional insertion of pieces of PGA (polyglycolic acid) sheet into ruptured bulla alternately.

## Methods

1) VATS is introduced with general anesthesia, 2) detect the responsible focus, 3) confirm that stapling is not good for the lesion, 4) ensure surgical margin to afford loop tie, 5) enlarge the air-leak hole or make a new hole to introduce a catheter, 6) FG infusion via a double lumen catheter, 7) a piece of PGA sheet insertion by endoscopic forceps, (repeat 6 and 7 a couple of times), 8) tie the catheter insertion site, 9) pleurodesis is added.

## Results

Since Oct. 2011 this operation was performed at our institution 9 times for 8 patients with uncontrolled secondary spontaneous pneumothoraces. Their underlying conditions were COPD in 5, interstitial pneumonia (IP) in 2 and non-tuberculous mycobacterium (NTM) in 1. The NTM case required ECMO support during the operation due to poor pulmonary function. An IP case received this operation twice metachronously for another lesion. Air-leak could be perfectly controlled after this procedure in all cases. Chest tube could be withdrawn within 5 days after surgery and no serious morbidity was occurred.

## Conclusions

This layered FG and PGA insertion technique into ruptured bulla, named “mille-feuille therapy”, encourages us to perform the VATS for the patients with refractory pneumothoraces. This seems feasible and improves patients quality of life.

